# Scalp Metastasis as the Initial Presentation of Pancreatic Adenocarcinoma: A Case Report and Review of the Literature

**DOI:** 10.7759/cureus.116015

**Published:** 2026-09-09

**Authors:** Hassane Ait Ali, Kharkhach Ayoub, Tariq Bouhout, Badr Serji

**Affiliations:** 1 Laboratory of Cancer Research and Health Sciences, Faculty of Medicine and Pharmacy of Oujda, Mohammed First University, Oujda, MAR

**Keywords:** cutaneous metastasis, immunohistochemistry, non-umbilical metastasis, pancreatic cancer, scalp metastasis

## Abstract

Cutaneous metastases from pancreatic ductal adenocarcinoma (PDAC) are rare and typically signify advanced disease with a poor prognosis. While umbilical involvement is the most frequently reported cutaneous manifestation, scalp metastases remain extremely unusual. This case report aims to describe an uncommon scalp metastasis from PDAC and to emphasize the importance of immunohistochemical analysis in atypical cases.

We report the case of a 71-year-old woman who presented initially with a painless nodule on her scalp and chronic epigastric discomfort, as well as unintentional weight loss, for at least six months. We conducted a thorough clinical examination, followed by contrast-enhanced computed tomography (CT) to stage the cancer systemically. We performed histopathological and immunohistochemical analyses on the excised scalp lesion to determine its origin.

Physical examination revealed epigastric tenderness, left supraclavicular lymphadenopathy, and an asymptomatic lesion on the scalp overlying the parietal bone. CT identified a primary tumor in the pancreatic body, with liver metastases and left supraclavicular lymphadenopathy. Immunohistochemical analysis revealed diffuse positivity for cytokeratin 7 (CK7) and cytokeratin 19 (CK19) and negative staining for CK20 and pP63. Therefore, the clinical, radiological, morphological, and immunohistochemical findings were consistent with pancreatic adenocarcinoma. The patient was subsequently diagnosed with Stage IV PDAC and commenced palliative combination chemotherapy with gemcitabine and nab-paclitaxel. At the four-month follow-up, she demonstrated radiographic stability and good local healing of the scalp biopsy site.

This case underscores the rarity of scalp metastasis from pancreatic adenocarcinoma and highlights the diagnostic utility of immunohistochemical markers in confirming atypical metastatic presentations. It also reinforces that persistent, enlarging, or otherwise suspicious scalp nodules require prompt evaluation and biopsy.

## Introduction

Pancreatic ductal adenocarcinoma (PDAC) constitutes the seventh leading cause of cancer-related mortality worldwide and has a five-year survival rate of only 10% [1]. The majority of patients are diagnosed at a locally advanced or metastatic stage, largely due to the lack of early clinical symptoms, which collectively portends a particularly dismal prognosis [1].

The most common sites of distant metastasis are the liver (79-94%), peritoneum (41-56%), abdominal lymph nodes (41%), and the lungs (45-48%) [2]. In contrast, cutaneous metastases are rare, occurring in < 1% of patients with pancreatic cancer and typically indicating widespread disease with poor survival outcomes. Although the Sister Mary Joseph umbilical nodule constitutes the most widely recognized cutaneous manifestation, involvement of other sites (including the lower extremities, trunk, and scalp) has been sporadically documented. Among these, scalp metastases remain extraordinarily rare, with only isolated cases reported in the existing literature [3].

Herein, we present an exceptional case of pancreatic adenocarcinoma that initially manifested as an asymptomatic scalp nodule accompanied by epigastric symptoms and supraclavicular lymphadenopathy. This case emphasizes the importance of clinicians maintaining an appropriate level of suspicion when confronted with persistent or otherwise suspicious scalp nodules, as they may indicate a systemic malignant tumor.

## Case presentation

A 71-year-old woman with no significant past medical history presented with a slowly growing nodule on her parietal scalp that had been present for more than six months. Her symptoms also included epigastric pain radiating to the back, unintentional weight loss of 5 kg, asthenia, insomnia, and anorexia. But the patient reported that the scalp nodule appeared before the other symptoms. She denied any jaundice, fever, or personal or family history of pancreatic or other malignancies and had no notable tobacco or alcohol use.

On physical examination, the patient had an Eastern Cooperative Oncology Group (ECOG) performance status of 2 [4]. A firm, mobile, non‑tender, 2‑cm nodule with a crusted, eroded surface and surrounding erythema was noted on the parietal scalp (Figure 1).

**Figure 1:**
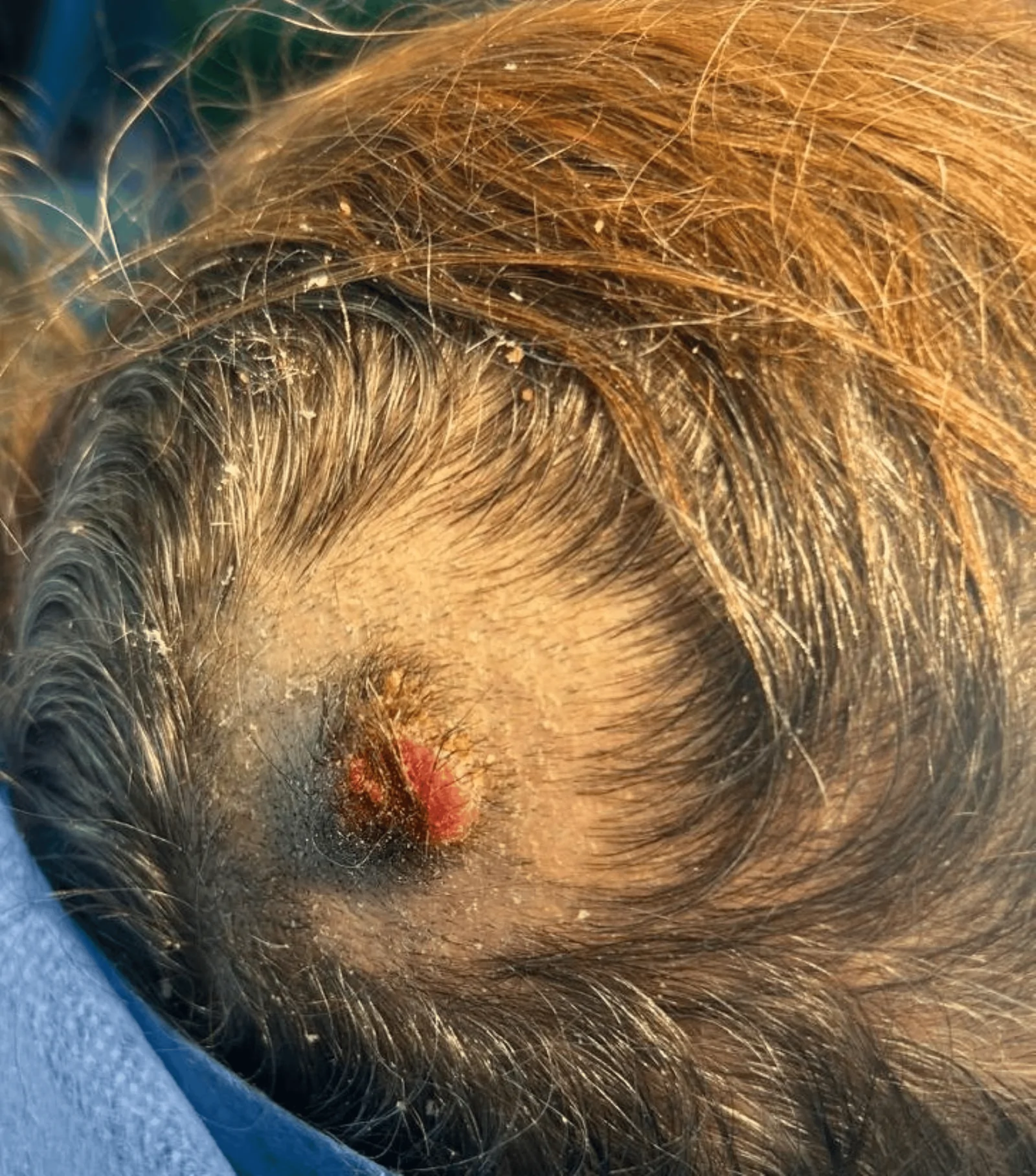
Photograph of lesion on the scalp (parietal area) Showing a firm, non-tender nodule measuring approximately 2 cm in diameter on the scalp (parietal area), with surface crusting and erosion, accompanied by erythema in the surrounding area.

However, palpation of the neck revealed an enlarged, firm left supraclavicular lymph node (Virchow's node), measuring approximately 2 cm in diameter, and abdominal examination revealed mild epigastric tenderness.

Laboratory investigations revealed normal liver enzymes and bilirubin levels. However, serum tumor markers were elevated, with a carbohydrate antigen 19-9 (CA19-9) level of 106 U/mL (normal <37 U/mL) and a carcinoembryonic antigen (CEA) level of 8.5 ng/mL (normal <5 ng/mL). Contrast-enhanced computed tomography (CT) scans of the abdomen, thorax, and brain revealed a 4.7 x 3.1 cm hypodense mass in the pancreatic body, multiple hypodense liver metastases, and left supraclavicular lymphadenopathy with a retropancreatic lymph node mass (Figure 2).

**Figure 2:**
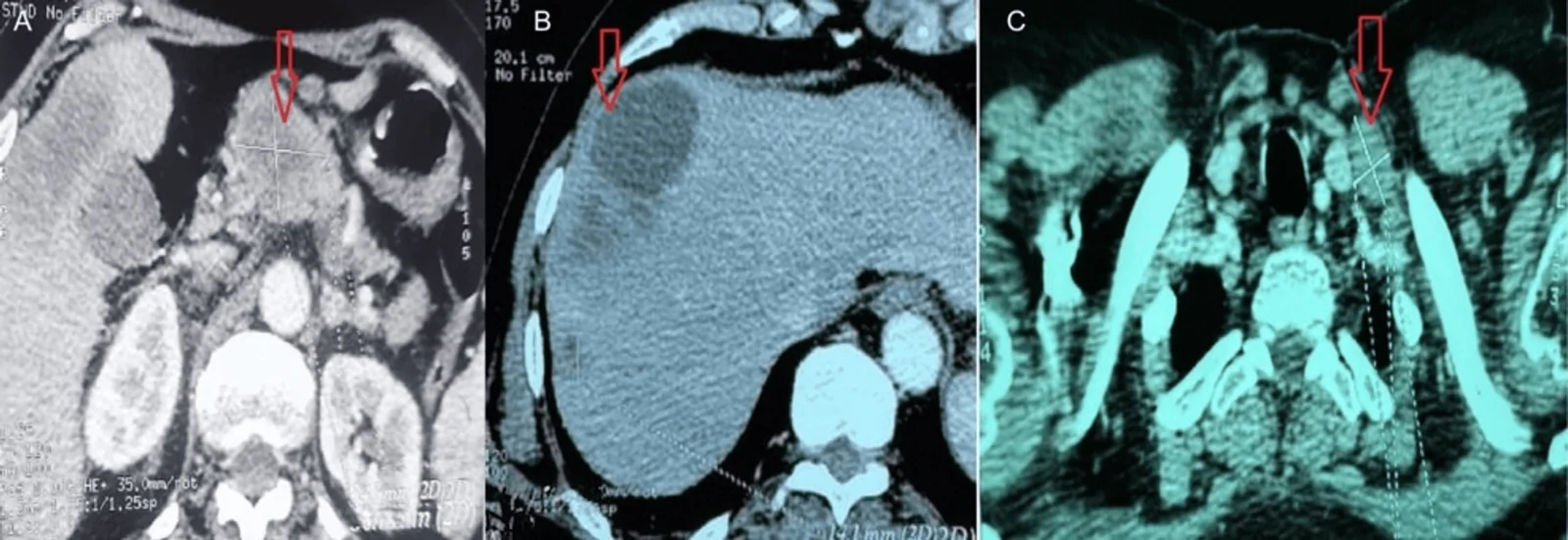
Contrast-enhanced computed tomography (CT) scan of the chest, abdomen, and pelvis A: a hypodense mass located in the body of the pancreas (red arrow); B: liver metastases (red arrow); C: left cervical lymph node (red arrow).

No pulmonary or cerebral metastatic lesions were detected. Given this unusual presentation, excisional biopsies of the parietal scalp nodule and the left supraclavicular lymph node were performed under local anesthesia with sedation. Histopathological examination of the scalp specimen revealed dermal infiltration by nests of poorly differentiated adenocarcinoma cells with pleomorphic nuclei, prominent nucleoli, and abundant eosinophilic cytoplasm (Figure 3).

**Figure 3:**
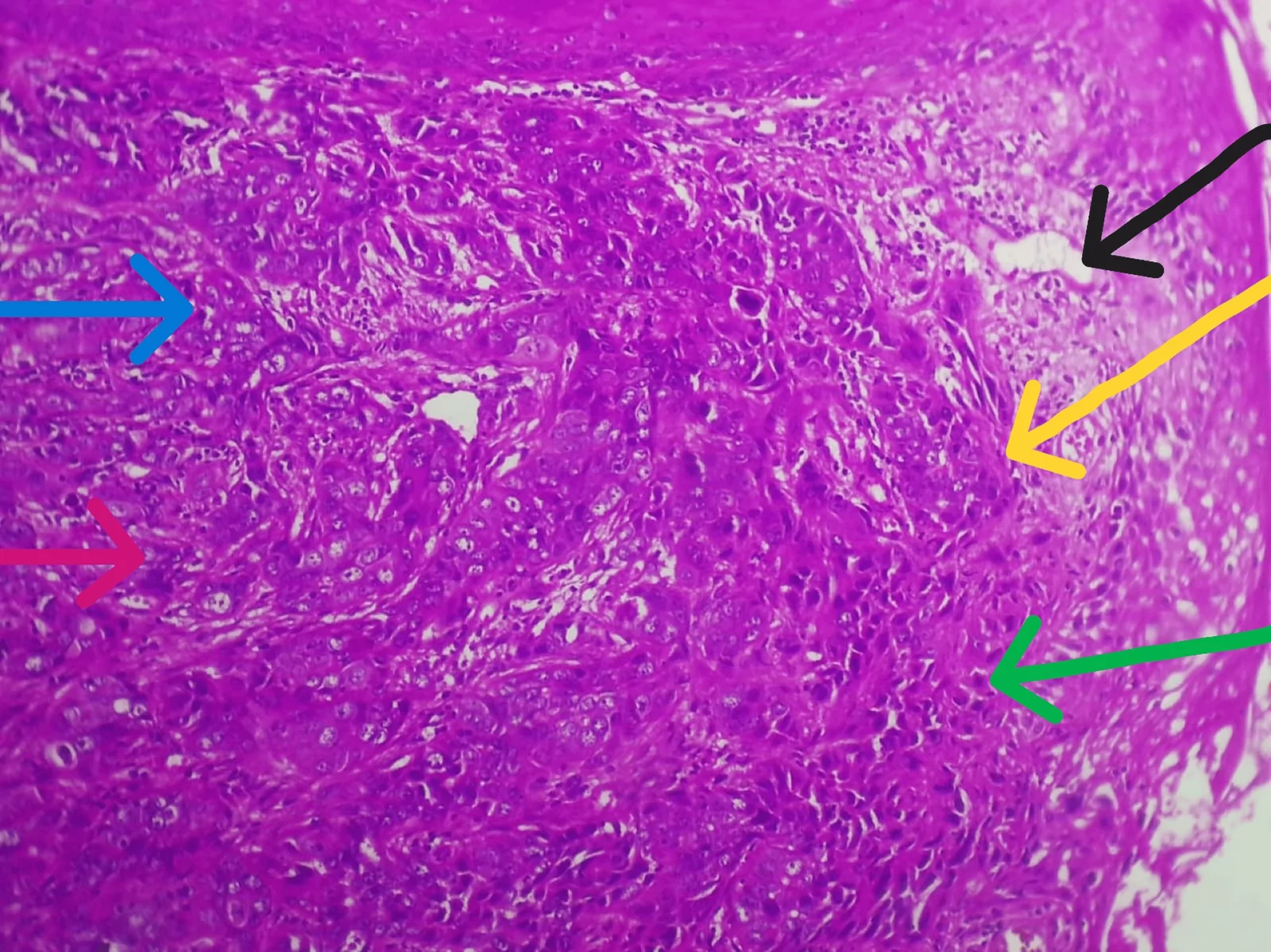
Microscopic examination of the excised scalp lesion This image shows dermal infiltration by nests of poorly differentiated adenocarcinoma (hematoxylin and eosin stain; original magnification ×100; scale bar = 100 µm). The tissue reveals extensive infiltration of the dermis by sheets of atypical cells (green arrow). These cells show marked nuclear pleomorphism, irregular nucleoli (blue arrow), and abundant cytoplasm (red arrow). Distinct glandular formations (yellow arrow) are observed, with some retaining a visible central glandular lumen (black arrow), confirming the adenocarcinoma nature.

Histopathological examination of the left supraclavicular lymph node specimen revealed effacement of the nodal architecture by metastatic, poorly differentiated adenocarcinoma with similar morphological features. The morphological appearance of the tumor cells was consistent between the scalp and lymph node specimens, indicating a shared primary origin.

Immunohistochemical staining was performed on both specimens to determine the primary origin. In the scalp lesion, the neoplastic cells demonstrated diffuse positivity for cytokeratin 7 (CK7) and cytokeratin 19 (CK19), with negative staining for CK20 and the transcription factor P63 (Figure 4).

**Figure 4:**
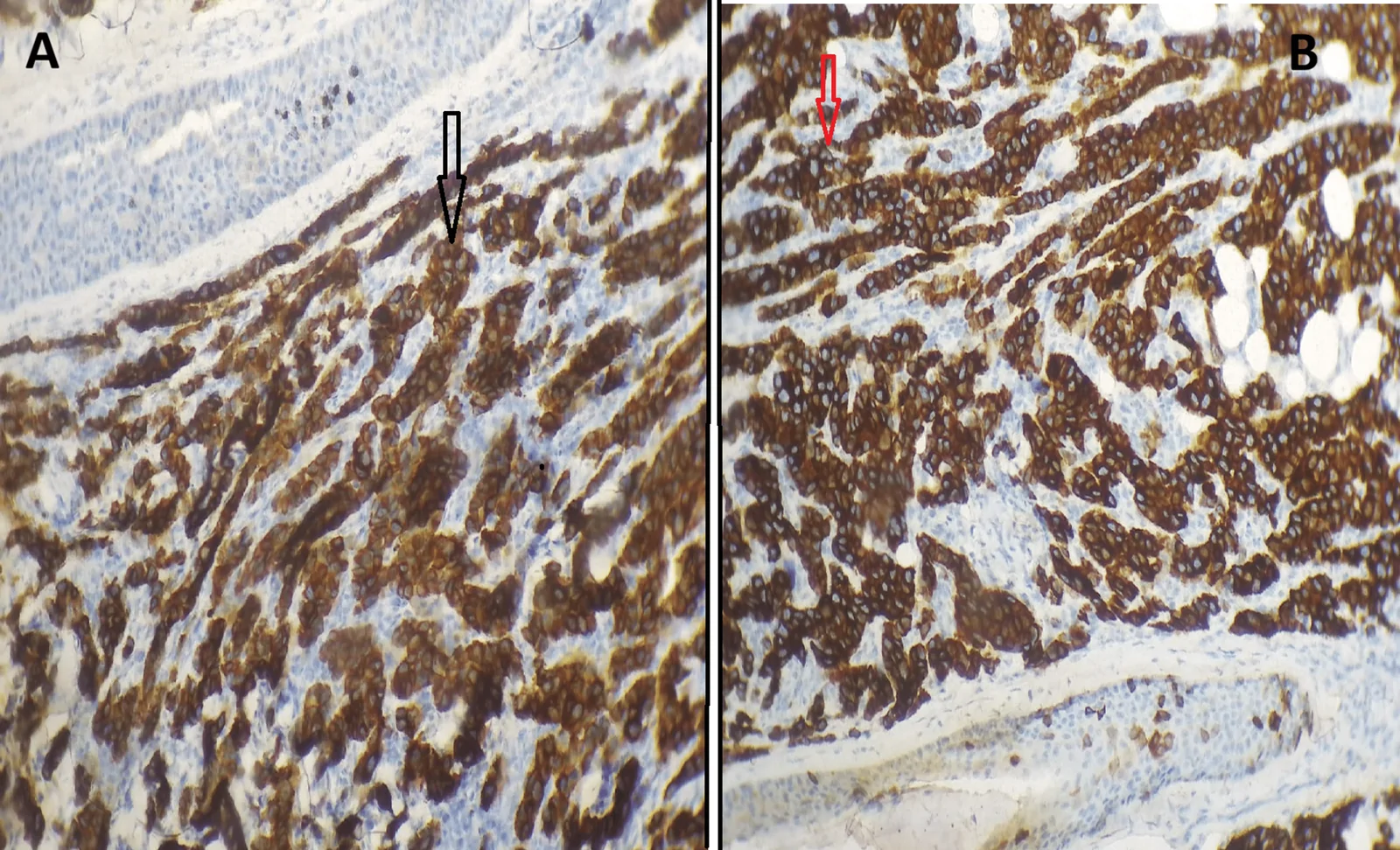
Immunohistochemical staining of the excised scalp lesion A: positive reaction of cytokeratin 7 (black arrow) (x100); B: positive reaction of cytokeratin 19 (red arrow) (x100). These markers suggest that the lesion has metastasized from an adenocarcinoma of pancreaticobiliary origin.

Immunohistochemical analysis of the supraclavicular lymph node specimen revealed concordant diffuse positivity for CK7 and CK19, with negative staining for CK20. These markers indicated that the lesion was a metastasis of pancreaticobiliary adenocarcinoma.

Based on these findings, with the presence of a pancreatic mass and hepatic metastases on imaging, the diagnosis is consistent with Stage IV PDAC (cT3N1M1), which has metastasized to the scalp, liver, and supraclavicular lymph nodes. Following a discussion at a multidisciplinary meeting, she was referred to the medical oncology department for palliative chemotherapy.

The patient began palliative systemic chemotherapy with gemcitabine (1000 mg/m²) and nab-paclitaxel (125 mg/m²) administered intravenously on days one, eight, and 15 of a 28-day cycle. At four-month follow-up, the patient has completed three cycles of therapy without any serious side effects.

Follow-up imaging with a CT scan demonstrated stable disease (SD) according to the RECIST 1.1 standards [5]. The surgical site on her scalp has healed well, showing no signs of recurrence. The patient continues to receive chemotherapy and supportive care for her symptoms.

## Discussion

This report describes an extremely rare presentation of metastatic PDAC, in which an asymptomatic scalp nodule constituted the first sign of Stage IV disease. PDAC is an aggressive cancer with a poor prognosis. This is due to its rapid progression and the frequent absence of early warning signs. Consequently, most patients present with locally advanced or metastatic disease. While distant dissemination most commonly involves the liver, peritoneum, lungs, bones, and brain [6], it can spread to all cutaneous tissues via the blood or lymphatic systems. Nevertheless, the precise molecular and mechanical mechanisms underlying such atypical metastatic patterns remain poorly understood. One hypothetical mechanism that has been proposed for metastases to unusual sites (including the scalp) involves hematogenous dissemination through the valveless vertebral venous plexus (Batson's plexus), which bypasses pulmonary capillary filtration and may facilitate retrograde spread to distant tissues [7]. However, it is important to emphasize that this mechanism has not been established for PDAC.

Cutaneous metastases are uncommon, occurring in only 0.7-9% of all cancer patients, and most frequently arise from the breast (50.5%), melanoma (18.3%), and colorectal cancer (4.3%) [8]. However, PDAC contributes to < 2% of all cutaneous metastases, with involvement of the scalp being an extremely rare occurrence [9]. Interestingly, cutaneous metastases are significantly more prevalent in cancers of the body and tail of the pancreas than in those of the head. This disparity is likely due to the silent clinical nature of body and tail cancers, which often progress to advanced and metastatic disease before becoming symptomatic [10]. We conducted a systematic literature review to contextualize this case within previously reported instances of pancreatic cancer scalp metastases, searching PubMed/Medical Literature Analysis and Retrieval System Online (MEDLINE) up to 15 August 2026. The search strategy used the terms: ("scalp metastasis" OR "scalp nodule" OR "scalp lesions") AND ("pancreatic adenocarcinoma" OR "pancreatic cancer" OR "pancreatic carcinoma" OR "pancreatic ductal adenocarcinoma" OR "pancreatic neoplasms"). No language restrictions were applied. We identified 12 eligible cases, which are summarized in Table 1.

**Table 1:** Summary of previously reported cases of scalp metastasis from pancreatic cancers M: male; F: female

Author (Year)	Age/Sex	Primary Site	Time to Scalp Metastasis	Concurrent Metastases	Treatment	Outcome
Miyahara M (1996) [11]	43/M	Uncus	Metachronous (15 months)	Not reported	Not reported	Not reported
Ambro CM (2006) [12]	63/M	Not reported	Synchrouns	Liver and lung	Chemotherapy	Alive at 8 months
Kaoutzanis C et al. (2013) [13]	43/M	Head	Synchronous	Peritoneum	Palliative chemotherapy	Died at 4 months
Bdeiri et al. (2013) [14]	70/F	Tail	Synchronous	Liver and peritoneum	Palliative chemotherapy	Died at 3 months
Zhou et al. (2014) [6]	76/F	tail	Synchrouns	Peritoneum lymph node, lung, liver, and additional cutaneous lesions	Palliative chemotherapy	2 months
Tavío-Hernández E (2015) [15]	81/F	uncus	Synchrouns	Lung, peritoneum, and muscles	Palliative care	Not reported
Moon JI et al. (2016) [16]	83/F	Head	Synchronous	Liver, adrenal gland, and bones	Not reported	Died at 4 months
Salari et al. (2020) [17]	66/M	Tail	Synchronous	Liver, lung, peritoneum and additional cutaneous lesions	Palliative chemotherapy	Died at 11 months
Ramachandran et al. (2020) [18]	76/M	Head	Synchronous	Muscles, subcutaneous tissues, bone, lung, spleen, liver, and colon.	Palliative care	Died at 06 weeks
Bashinskaya et al. (2023) [19]	61/M	Not reported	Metachronous	Only on the scalp	Palliative chemotherapy	Alive
Nichkaode et al. (2024) [3]	59/F	Head	Synchronous	Retroperitoneal lymph nodes, bilateral adrenal glands, lung and liver	Palliative chemotherapy	Alive at 3 months
Posado-Domínguez L (2024) [9]	79/F	tail	Synchronous	Carcinomatous lymphangitis	Palliative care	Died at 17 days
Present case (2026)	71/F	Body	Synchronous	Liver, supraclavicular lymph node	Palliative chemotherapy (gemcitabine + nab-paclitaxel)	Stable at 04 months

Clinically, cutaneous metastases from PDAC typically present as firm, painless, mobile, or fixed subcutaneous nodules with normal overlying skin. These lesions may be solitary or multiple and can develop at any anatomical site. Due to their nonspecific appearance, they are often confused with benign dermatological conditions such as epidermoid cysts, lipomas, or dermatofibromas, which can delay diagnosis [10]. Consequently, this case underscores the need for clinicians to maintain a high index of suspicion and to pursue prompt histopathological assessment of any suspicious cutaneous lesion, particularly when accompanied by constitutional or abdominal symptoms, as it may represent the first or only overt sign of an underlying visceral malignancy.

A histopathological examination supported by immunohistochemical analysis is indispensable for confirming a diagnosis and excluding alternative primary sources. In cases of PDAC, the typical immunophenotype comprises diffuse positivity for CK7, CK19, CA19-9, and CEA, alongside negative staining for CK20, Caudal-type Homeobox 2 (CDX2), and thyroid transcription factor-1 (TTF-1), which supports the diagnosis of adenocarcinoma [20]. The following markers were not performed on the patient's specimens: TTF-1, Napsin A, CDX2, GATA3, estrogen receptor, and PAX8. Therefore, pulmonary, colorectal, breast, ovarian, and other alternative primary sites were not definitively excluded by immunohistochemistry alone. However, the diagnosis of pancreaticobiliary origin is supported by the integrated clinical, radiological, and pathological findings. Based on this integrated evidence, the diagnosis is most consistent with metastatic PDAC. Furthermore, the absence of the transcription factor p63 immunoreactivity in the present case helped distinguish the lesion from primary cutaneous adnexal tumors.

The current treatment options for metastatic or locally advanced PDAC are dependent on performance status (PS) [21]. In patients with an ECOG PS of 0-1, a bilirubin level of <1.5 times the upper limit of normal, and no major comorbidities, two regimens should be recommended as first-line chemotherapy: FOLFIRINOX or NALIRIFOX. Gemcitabine-nab-paclitaxel can also be used in these patients. In patients with ECOG PS 2, Karnofsky performance status ≥ 70, and bilirubin ≤ 1.5 × upper limit of normal, gemcitabine-nab-paclitaxel can be considered.

Unfortunately, first-line chemotherapy regimens confer only modest improvements in overall survival, with most patients failing to survive beyond one year [6]. The median survival time from the diagnosis of cutaneous metastasis is reported to be five months [8]. This reality underscores the importance of recognizing atypical metastatic presentations and accurately characterizing them histopathologically. While the identification of Stage IV disease through cutaneous involvement is prognostically unfavorable, it enables the timely initiation of palliative care and appropriate oncological surveillance. Ultimately, this case reaffirms that seemingly insignificant scalp nodules can indicate visceral carcinomas and highlights the enduring value of comprehensive physical examination and immunohistochemical analysis.

## Conclusions

In summary, this case illustrates a very rare instance of PDAC that initially manifested as a solitary cutaneous metastasis in the scalp. Although this finding is considered a poor prognostic sign, it shows that scalp lesions can indicate visceral malignancy.

This report reinforces the indispensable role of immunohistochemistry in diagnosis and management. Although the treatment options for metastatic PDAC remain limited, with current chemotherapy regimens providing only modest survival benefits, prompt identification through cutaneous biopsy enables the initiation of palliative care and appropriate oncological surveillance. Therefore, clinicians should maintain a high level of suspicion for metastatic disease when atypical cutaneous nodules, accompanied by abdominal symptoms, weight loss, lymphadenopathy, or other findings, raise suspicion of systemic malignancy.
